# Tolerance to alternative cyclooxygenase-2 inhibitors in nonsteroidal anti-inflammatory drug hypersensitive patients

**DOI:** 10.1186/2045-7022-3-20

**Published:** 2013-06-24

**Authors:** Wendy SJ Malskat, André C Knulst, Carla AFM Bruijnzeel-Koomen, Heike Röckmann

**Affiliations:** 1 Department of Dermatology/Allergology, University Medical Centre Utrecht, Heidelberglaan 100, G 02.124, Utrecht 3584 CX, The Netherlands; 2 Current Address: Department of Dermatology, Erasmus Medical Centre, Rotterdam, The Netherlands

**Keywords:** COX-2, Drug hypersensitivity, Nonsteroidal anti-inflammatory drugs, Oral provocation

## Abstract

**Background:**

Non-steroidal anti-inflammatory drugs (NSAIDs) frequently cause adverse drug reactions. Many studies have shown that drugs which selectively inhibit the cyclooxygenase-2 enzyme (COX-2) are safe alternatives in the majority of patients. However, hypersensitivity reactions to COX-2 inhibitors have been published. Hardly any data are available regarding the safety of alternatives in case of COX-2 inhibitor hypersensitivity. We aimed to investigate the tolerance to COX-2 inhibitors in patients with non-selective NSAID hypersensitivity. Furthermore, in COX-2 hypersensitive patients tolerance of a second COX-2 inhibitor was investigated.

**Methods:**

We retrospectively analyzed 91 patients with proven non-selective NSAID hypersensitivity that underwent oral challenges with a COX-2 inhibitor. Patients with intolerance to the first challenged COX-2 inhibitor received a second challenge with a different COX-2 inhibitor.

**Results:**

19 out of 91 (21%) patients had a positive reaction to the first oral challenge with a COX-2 inhibitor. 14 of them underwent a second challenge with a different COX-2 inhibitor and 12 (86%) did not react.

**Conclusions:**

A relatively high percentage (21%) of the non-selective NSAID hypersensitive patients did not tolerate a COX-2 inhibitor and oral challenge is advised prior to prescription of a COX-2 inhibitor. For the majority of patients reacting to a COX-2 inhibitor an alternative can be found.

## Background

NSAIDs (non-steroidal anti-inflammatory drugs) are the most universally used analgesics and are responsible for about 21-35% of all drug hypersensitivity reactions [1-3]. Symptoms vary from cutaneous (urticaria and/or angioedema) and respiratory (rhinitis and/or dyspnea) to anaphylactic shock. The frequency of NSAID hypersensitivity might be higher in patients with chronic spontaneous urticaria, asthma [4-6] and mastocytosis [7]. The majority of reactions (about 75%) are not caused by immunological mechanisms (IgE or T-cell mediated), but by pharmacological inhibition of the cyclooxygenase-(COX) pathway [8]. This is supported by the clinical observation that many NSAID hypersensitive patients react to various NSAIDs of unrelated structure [5,6,9-11]. There are at least 2 isoforms of COX; the constitutively expressed COX-1 enzyme takes part in fundamental mechanisms of homeostasis, whereas the inducible COX-2 enzyme mediates inflammation. The therapeutic effects of NSAIDs are thought to be mainly related to the inhibition of COX-2, whereas COX-1 inhibition seems to be more responsible for the adverse effects [6,10]. For that reason selective COX-2 inhibitors have been developed.

Therapeutic options to diagnose NSAID hypersensitivity are still limited. Oral drug challenge (preferably placebo controlled) is the gold standard in diagnosing NSAID hypersensitivity [12,13].

Several studies have shown (partial) tolerability of COX-2 inhibitors in patients with hypersensitivity to non-selective NSAID and/or aspirin sensitive asthma [14-16]. Weberstock et al. [15] reviewed 84 studies, 13 of them described double-blind COX-2 inhibitor challenges. Of the 3304 patients described, 119 (3.6%) had hypersensitivity to COX-2 inhibitors, consisting of urticaria, angioedema and/or rhinorrhea. Therefore, NSAIDs that selectively inhibit COX-2 are assumed to be a safe alternative in the majority of non-selective NSAID hypersensitive patients. A recent review by Asero [17] described a wide variation in the percentage of COX-2 inhibitor hypersensitivity up to 33%. Recently, Dona et al. [18] described a high percentage of COX-2 inhibitor hypersensitivity (25%) in patients with hypersensitivity to multiple non-selective NSAIDs and paracetamol. In the same study, patients hypersensitive to multiple non-selective NSAIDs, but tolerant to paracetamol showed only 6% of COX-2 inhibitor hypersensitivity.

So far, tolerance of an alternative COX-2 inhibitor in COX-2 hypersensitive patients has been hardly investigated. A case-report of 2 patients with hypersensitivity to numerous non-selective NSAIDs and a selective COX-2 inhibitor, described tolerance to a second COX-2 inhibitor, celecoxib and etoricoxib respectively [19]. Quinones Estevez [20] described a case series of 8 patients with hypersensitivity to non-selective NSAIDs and selective COX-2 inhibitors. Three of 5 patients that were challenged tolerated an alternative COX-2 inhibitor; celecoxib or etoricoxib. A study comparing tolerance to different COX-2 inhibitors (n = 37) (nimesulide, meloxicam and rofecoxib) showed tolerance to meloxicam in 8 of 11 patients hypersensitive to nimesulide. The majority of nimesulide hypersensitive patients (10/11) tolerated rofecoxib [21].

The aim of this study was to assess the tolerance of a first and, in case of intolerance, a second COX-2 inhibitor in patients with hypersensitivity to non-selective NSAIDs in a larger population.

## Methods

### Selection of patients

All patients (n = 91) with proven non-selective NSAID hypersensitivity and oral challenge to a selective COX-2 inhibitor at the outpatient clinic of Allergology of the University Medical Center Utrecht, from September 2002 until April 2012, were analyzed. NSAID hypersensitivity was diagnosed, based on either a convincing unequivocal patient history (n = 69), or a positive oral challenge with the suspicious drug (n = 22). Challenge protocols are shown in Table 1. The criteria for a convincing patient history [22] were: 1. a time interval of a few minutes up to a maximum of 5 hours between intake of the drug and start of symptoms, 2. objective signs of urticaria, angioedema, rhinitis, dyspnea and/or anaphylactic shock (systolic BP <90 mm Hg or a >30 mm Hg drop). We analyzed if patients had two or more reactions to the same or distinct NSAID(s), reported either by challenge or by history. We also analyzed usage and intolerance of paracetamol after reaction to the culprit drug documented in patient history.

**Table 1:** Challenge protocols of culprit NSAIDs

	**Diclofenac**	**Ibuprofen**	**Aspirin**	**Naproxen**	**Propyfenazon**
**Administration**	oral	oral	oral	oral	oral
**Dose 1 (mg)**	0.1	0.1	10	0.1	1
**Dose 2 (mg); time interval (min)**	1; 30	1; 30	44; 30	1; 30	5; 30
**Dose 3 (mg); time interval (min)**	10; 30	10; 30	117; 60	10; 30	25; 30
**Dose 4 (mg); time interval (min)**	25; 60	50; 30	312; 60	50; 30	100; 60
**Dose 5 (mg); time interval (min)**	50; 60	100; 60	500; 120	125; 30	250; 60
**Dose 6 (mg); time interval (min)**	NA	200; 60	NA	250; 60	NA

**Legend:***Min*, minutes; *NA*, not applicable; (Since this study the dosages have been revised and the dosage of 0.1 mg is removed.)

### Study design

Sex, age, suspected NSAID(s) and co-morbidity data were obtained, using patient files and, where necessary, telephone calls to complete and confirm the data. The clinical reaction patterns, the challenge with the culprit drug and the outcome of challenge with COX-2 inhibitor were assessed based on differentiated organ involvement: cutaneous (urticaria and/or angioedema), respiratory (rhinitis and/or dyspnea) or anaphylactic shock. The Medical Ethics Review Committee (METC) of UMC Utrecht agreed to the study (METC-protocol number 12-438).

### COX-2 inhibitor challenges

At first COX-2 inhibitor challenges were performed with rofecoxib, until it was withdrawn from the market in 2003 (dosages: 1, 6.25, and 12.5 mg; interval 60 minutes). After 2003, celecoxib was preferentially used for a first challenge (dosages: 0.1, 1, 10, 20 mg at 30 minutes intervals and 50 and 100 mg at 60 minutes intervals). Etoricoxib was preferentially used for a second challenge (dosages: 0.1, 1, 5, 10 mg at 30 minutes intervals and 30 and 60 mg at 60 minutes intervals). The time interval between different oral challenges was 6–12 weeks.

The challenges were performed in a clinical setting, equipped for resuscitation and monitoring of vital signs. Appearance of symptoms was closely monitored. A challenge test was considered to be positive when objective symptoms occurred, e.g. itching and erythema, urticaria, angioedema, rhinoconjunctivitis, dyspnea, hypotension or anaphylactic shock. If objective symptoms were present, the challenge was stopped and the symptoms were treated with antihistamines or prednisone. None of the patients required treatment with epinephrine.

### Statistical analysis

Numeric results were expressed as mean ± standard deviation (SD). Nominal variables were expressed as percentage of the patients. The Statistical Package for Social Sciences (SPSS) for Windows Version 15.0 was used to analyze the data.

## Results

### Patient characteristics

91 patients with a diagnosis of non-selective NSAID hypersensitivity were challenged with a first COX-2 inhibitor. The demographic and clinical characterizations of the study group, as well as the drugs suspected to cause a hypersensitivity reaction are shown in Table 2 and 3. Diagnosis of NSAID hypersensitivity was confirmed by oral challenge with the culprit drug in 22 patients. In 69 patients, the history was highly suggestive and no oral challenges were performed. 44 (of 69) patients (63%) reported repeated reactions to the same culprit drug. Of the 22 patients with a single reaction and without challenge to the culprit drug 11 patients (50%) experienced anaphylactic reactions. In 3 patients information about repeated reactionswas missing.

**Table 2:** Demographic and clinical features of the study group

**Variable**	**Patients (n = 91)**
Sex (male/female) n (%)	**23/68 (25/75%)**
Age (years) (mean ± SD)	**51.9 ± 13.3**
Reaction to 1 NSAID n (%)	**71 (78%)**
Reaction to various NSAIDs n (%)	**20 (22%)**
Reaction to paracetamol^1^	**17 (18%)**
Co-morbid disorders n (%)	
- Chronic spontaneous urticaria	**11 (12%)**
- Asthma	**9 (10%)**
Diagnosed non-selective NSAID hypersensitivity n (%)	
- Patient history; repeated reaction to the same culprit drug	**44 (48%)**
- Patient history; no/unknown repeated reaction to the same culprit drug	**25 (28%)**
- Oral challenge with the culprit drug	**22 (24%)**

^1^by patient history.

**Table 3:** Type of reaction to the suspected drugs according to patient history

**Specific drug**	**Type of reaction**				
	**Urticaria**^**1**^	**Angioedema**^**2**^	**Respiratory**^**3**^	**Anaphylaxis**^**4**^	**Total n (%)**
Diclofenac	6	4	10	22	42 (46%)
Ibuprofen	2	5	4	4	15 (16%)
Naproxen	1	3	2	2	8 (9%)
Aspirin	0	2	2	0	4 (4%)
Propyfenazon	0	1	1	0	2 (2%)
Various NSAIDs	6	8	2	4	20 (22%)
Total	15 (16%)	23 (25%)	21 (23%)	32 (35%)	91 (100%)

^1^Presence of urticaria (without angioedema).

^2^Presence of angioedema (with or without urticaria).

^3^Presence of respiratory symptoms (rhinitis, dyspnea) (additional to urticaria and/or angioedema).

^4^Presence of hypotension <90 mmHg or >30 mmHg drop (additional to urticaria and/or angioedema).

Of the 91 patients, 9 (10%) suffered from concomitant chronic asthma and 11 patients (12%) from concomitant chronic spontaneous urticaria unrelated to the intake of NSAIDs. The majority of the patients (n = 71; 78%) reported a reaction to one NSAID, while 22% (n = 20) reacted to several (2 or 3) non-selective NSAIDs. Paracetamol was tolerated in 70 patients (79%) following reaction to a NSAID. Information on paracetamol tolerance was missing in 4 patients. The most frequently involved drug was diclofenac in 42 patients (46%). Anaphylactic shock was the most frequent reaction type reported in 32 subjects (35%), followed by angioedema in 23 subjects (25%). None of the patients suffered from aspirin sensitive asthma.

### First COX-2 inhibitor challenges

In 77 patients (85%) celecoxib was given and in 13 patients (14%) rofecoxib. Etoricoxib was used in the first challenge in only one patient. 19 patients (21%) had a positive challenge to one of these COX-2 inhibitors. Characteristics and symptoms of patients with a positive first challenge are presented in Table 4 and 5. Three of these COX-2 hypersensitive patients suffered from concomitant chronic spontaneous urticaria but were free of urticarial symptoms for 6 weeks or more, before oral challenge with the COX-2 inhibitor. In 14 of 19 patients (74%) diagnosis of non selective NSAID hypersensitivity was confirmed by challenge or repeated reaction to the culprit NSAID. Eight of the 19 patients (42%) with a positive challenge to a COX-2 inhibitor had a history of hypersensitivity to multiple non-selective NSAIDs. Four patients were intolerant to paracetamol. Ten patients (16%) with unknown status of hypersensitivity to multiple non-selective NSAIDs, reacted to a COX-2 inhibitor.

**Table 4:** Demographic and clinical features of patients with positive first COX-2 inhibitor challenge

**Variable**	**Patients (n = 19)**
Sex (male/female) n (%)	**4/15 (21/79%)**
Age (years) (mean ± SD)	**53.2 ± 10.9**
Reaction to 1 NSAID n (%)	**12 (63%)**
Reaction to various NSAIDs n (%)	**7 (37%)**
Reaction to paracetamol^1^	**4 (21%)**
Co-morbid disorders n (%)	
- Chronic spontaneous urticaria	**3 (16%)**
- Asthma	**4 (21%)**
Diagnosed non-selective NSAID hypersensitivity n (%)	
- Patient history; repeated reaction to the same culprit drug	**11 (58%)**
- Patient history; no/unknown repeated reaction to the same culprit drug	**3 (15%)**
- Oral challenge with the culprit drug	**5 (26%)**

^1^By patient history.

**Table 5:** Clinical features and results of patients with positive first COX-2 inhibitor challenge

**Patient no.**	**Sex/age**	**Co-morbid disorders**	**Culprit drug**	**Paracetamol intolerance**	**Type of reaction**	**Manner of diagnosis**	**Reaction to oral COX-2 inhibitor challenges**			
							**First**		**Second**	
							**Drug Reaction**		**Drug Reaction**	
1	F/55	As	diclofenac	no	U/A/Ana	PH; repRX	rofecoxib	U/D	nd	
2	M/78	NS	diclofenac	no	A/D/Ana	C	rofecoxib	U	nd	
3	F/65	As	multiple	no	U	PH; repRX	celecoxib	U	rofecoxib	Neg
4	F/55	NS	diclofenac	no	U/Ana	C	rofecoxib	A	celecoxib	Neg
5	F/46	NS	naproxen	no	A	PH	rofecoxib	A	celecoxib	Neg
6	M/36	NS	multiple	yes	U/A	PH; repRX	celecoxib	U	nd	
7	F/35	NS	multiple	no	U/A	C	etoricoxib	U/A	celecoxib	Neg
8	F/51	As	diclofenac	no	A/D	PH; repRX	celecoxib	U/An/D/A	nd	
9	F/56	NS	multiple	yes	A	C, repRX	celecoxib	A	rofecoxib	Neg
10	F/50	NS	naproxen	no	U/Ana	PH; repRX	celecoxib	A	etoricoxib	Neg
11	F/59	NS	naproxen	no	U/A	PH; repRX	celecoxib	A	etoricoxib	Neg
12	F/46	NS	diclofenac	no	U/A/D/Ana	PH	celecoxib	A	etoricoxib	Neg
13	F/62	U	multiple	yes	U/A	PH; repRX	celecoxib	U	nd	
**14**	**M/40**	**U**	**ibuprofen**	**yes**	**U/A**	**PH; repRX**	celecoxib	**U/D**	etoricoxib	**U/D**
15	F/66	NS	diclofenac	no	U/A/D/Ana	PH; repRX	celecoxib	U/A	etoricoxib	Neg
16	F/63	NS	multiple	no	U/A/ D	PH; repRX	celecoxib	U/A	etoricoxib	Neg
**17**	**F/53**	**NS**	**diclofenac**	**no**	**U**	**C**	celecoxib	**U/A**	etoricoxib	**U/A/D**
18	F/46	A/U	multiple	no	U/A	PH; repRX	celecoxib	A	etoricoxib	Neg
19	M49	NS	diclofenac	no	U/A	PH	celecoxib	U/A	etoricoxib	Neg

**Legend**: *F*, female; *M*, male; *NS*, none specific; *As*, asthma; *U*, urticaria; *A*, angioedema (without weals); *Ana*, anaphylactic shock; *D*, dyspnea; *PH*, patient history; *repRx*, repeated reaction to the same drug; *C*, challenge test; *nd*, not done; *Neg*, negative reaction. 2 patients with a positive challenge to a second COX-2 inhibitor are highlighted in **bold.**

### Second COX-2 inhibitor challenges

14 out of 19 patients with a positive first challenge to a COX-2 inhibitor underwent a second challenge test with a different COX-2 inhibitor (9 etoricoxib, 3 celecoxib and 2 rofecoxib), while 5 patients declined. The second provocation was positive in only 2 patients. Both patients presented with urticaria, generalized itch and dyspnoea following challenge with etoricoxib. One of them had a history of hypersensitivity reactions to the non-selective NSAIDs ibuprofen and naproxen together with a past history of chronic spontaneous urticaria, but not with asthma. The second patient had a history of hypersensitivity reactions to diclofenac, without chronic spontaneous urticaria or asthma.

## Discussion

In this retrospective study a relative high percentage of 21% COX-2 hypersensitivity was found in patients with non-selective NSAID hypersensitivity. However, in COX-2 hypersensitive patient’s tolerance to another COX-2 inhibitor was found in the majority (86%) of patients.

There is a large variation (0% to 33%) in the percentage of COX-2 inhibitor hypersensitivity in patients with NSAID hypersensitivity (17). Most studies describe low percentages [15,21,23-37]. One study associated a high percentages (25%) of hypersensitivity to etoricoxib with paracetamol intolerance (compared to 6% in multiple NSAID hypersensitivity without paracetamol intolerance) [18]. In contrast, COX-2 inhibitor hypersensitivity was not associated with paracetamol hypersensitivity in our patient population.

Differences in study design may explain the variability in the different studies. Many studies did not perform oral challenges to confirm non selective NSAID hypersensitivity [21,24,26,27,29,34,35,38]. This may have induced a higher percentage of incorrect diagnosis of non selective NSAID hypersensitivity, especially since some studies included patients with a long time interval, up to 72 hours, between intake of the culprit non selective NSAID and onset of symptoms [26,29,33]. Incorrect diagnosis of non selective NSAID hypersensitivity may consequently lead to a lower estimate of the percentage of COX-2 inhibitor hypersensitivity. The majority of patients in our study were also included based on a suggestive history. However, we used strict criteria: only patients with objective symptoms and within 5 hours after intake were included. Furthermore, 53 (58%) patients had re-intake of the same culprit drug followed by a similar hypersensitivity reaction, which strongly supports the diagnosis. 44 patients with a repeated reaction did not underwent a challenge with the culprit drug. In addition, the percentages of COX-2 hypersensitivity did not differ between the patients diagnosed by careful history only versus those in which the diagnosis was confirmed by challenge.

Provocation with COX-2 inhibitors was open and not placebo controlled. However, the challenges were only considered positive if the patient developed objective symptoms, in agreement with other studies [21,36]. Since we performed this study, the challenged drug dosages have been revised; the lowest dosage of 0.1 mg has appeared to be unnecessary and has been removed from the protocol.

Another important factor that might influence the rate of COX-2 inhibitor hypersensitivity is patient selection. Patients in our population who previously reacted to numerous non-selective NSAIDs had a higher rate (38%) of reaction to COX-2 inhibitors than patients who reacted to only one NSAID (16%). This is in line with Dona et al., analyzing patients with multiple NSAID hypersensitivity, showing a generally high percentage of hypersensitivity to etoricoxib [18]. Multiple NSAID reactivity was not known in all patients. Therefore, the current classification of EAACI/ENDA group [11] could not be applied to subdivide the study population.

Another important factor could be the specific culprit drug, since acetylsalicylic acid (ASA) was the most frequently reported culprit drug in many other studies (21 - 100%) [14,21,23,25-27,29,30,33-36,39],[40]. In our study population, diclofenac, ibuprofen and naproxen represented the most common culprit drugs with 46% , 15% and 9% , respectively. Only 4 patients (4%) reported a hypersensitivity reaction to ASA only. None of them showed a reaction to a COX-2 inhibitor. Further studies are needed to investigate the relation between the specific culprit drug in non-selective NSAID hypersensitive patients and hypersensitivity to COX-2 inhibitors.

The frequency of chronic spontaneous urticaria (12%) in our study group was comparable with previous literature (2.7-11%) [21,23,29,41] and cannot explain the high percentage of COX- hypersensitivity. The frequency of asthma in our study population was rather low, compared to the literature [14,21,23,27,29,39]. Specific studies are needed to investigate whether hypersensitivity to COX-2 inhibitors is dependent on these factors.

Interestingly, a second COX-2 inhibitor was tolerated in the majority of challenged patients (86%). Of the patients with a second COX-2 inhibitor challenge, six had a history of reactions to multiple NSAIDs. However, five of them tolerated the challenge with a second COX-2 inhibitor. This shows that patients who react to a particular COX-2 inhibitor, may be tolerant to another one. This was also described in two small case-series [19,42]. Cimbollek et al. described two patients with multiple NSAID hypersensitivity reacting to one COX-2 inhibitor but tolerating another. Quinones-Estevez described 8 patients with multiple NSAID hypersensitivity of which five were challenged with both celecoxib and etoricoxib. Three patients reacted to only one of the drugs, while two to both. In our study, etoricoxib was mainly used as a secondly challenged alternative COX-2 inhibitor, known to have a much higher COX-2 selectivity than celecoxib [43]. This might explain the higher percentage of tolerance to etoricoxib. However, advantage of etoricoxib over celecoxib was not observed in case-series [19,20] or a recent review analyzing COX-2 inhibitor hypersensitivity [17].

Our results in a large group of patients with NSAID hypersensitivity and reaction to a specific COX-2 inhibitor show that an alternative COX-2 inhibitor can be tolerated.Challenge with a second COX-2 inhibitor can be recommended in these patients. So far, there seems no preference which COX-2 inhibitor, celecoxib or etoricoxib, should be challenged first and which second.

## Conclusions

In most patients with non-selective NSAID hypersensitivity COX-2 inhibitors were safe. However, given the relatively high percentage (21%) of positive challenges, it is necessary to perform an oral challenge prior to prescription. In case of hypersensitivity to a COX-2 inhibitor, second challenges with a different COX-2 inhibitor can provide an alternative. Further studies are needed to define risk factors for COX-2 inhibitor hypersensitivity in patients with non-selective NSAID hypersensitivity.

## Competing interests

The authors declare that they have no competing interests.

## Authors’ contributions

WM wrote the first version of the manuscript. WM and HR were involved in collection and interpretation of the data. HR, CB and AK contributed to the preparation and critical revision of the manuscript. All authors approved the final version of the manuscript.
